# Interacting factors associated with Low antibiotic prescribing for respiratory tract infections in primary health care – a mixed methods study in Sweden

**DOI:** 10.1186/s12875-016-0494-z

**Published:** 2016-07-18

**Authors:** Eva Lena Strandberg, Annika Brorsson, Malin André, Hedvig Gröndal, Sigvard Mölstad, Katarina Hedin

**Affiliations:** Department of Clinical Sciences, Malmö, Family Medicine, Lund University, Malmö, Sweden; Blekinge Centre of Competence, Blekinge County Council, Karlskrona, Sweden; Center for Primary Health Care Research, Malmö, Skåne Region Sweden; Department of Medicine and Health Sciences, Family Medicine, Linköping University, Linköping, Sweden; Department of Public Health and Caring Sciences – Family Medicine and Preventive Medicine, Uppsala University, Uppsala, Sweden; Department of Sociology Uppsala, Uppsala University, Uppsala, Sweden; Department of Research and Development, Region Kronoberg, Växjö, Sweden

**Keywords:** Mixed methods design, Antibiotic prescribing, Guidelines, Implementation, Primary care

## Abstract

**Background:**

Prescribing of antibiotics for common infections varies widely, and there is no medical explanation. Systematic reviews have highlighted factors that may influence antibiotic prescribing and that this is a complex process. It is unclear how factors interact and how the primary care organization affects diagnostic procedures and antibiotic prescribing. Therefore, we sought to explore and understand interactions between factors influencing antibiotic prescribing for respiratory tract infections in primary care.

**Methods:**

Our mixed methods design was guided by the Triangulation Design Model according to Creswell. Quantitative and qualitative data were collected in parallel. Quantitative data were collected by prescription statistics, questionnaires to patients, and general practitioners’ audit registrations. Qualitative data were collected through observations and semi-structured interviews.

**Results:**

From the analysis of the data from the different sources an overall theme emerged: A common practice in the primary health care centre is crucial for low antibiotic prescribing in line with guidelines. Several factors contribute to a common practice, such as promoting management and leadership, internalized guidelines including inter-professional discussions, the general practitioner’s diagnostic process, nurse triage, and patient expectation. These factors were closely related and influenced each other. The results showed that knowledge must be internalized and guidelines need to be normative for the group as well as for every individual.

**Conclusions:**

Low prescribing is associated with adapted and transformed guidelines within all staff, not only general practitioners. Nurses’ triage and self-care advice played an important role. Encouragement from the management level stimulated inter-professional discussions about antibiotic prescribing. Informal opinion moulders talking about antibiotic prescribing was supported by the managers. Finally, continuous professional development activities were encouraged for up-to-date knowledge.

## Background

Prescribing of antibiotics for common infections varies widely between different countries in Europe, and Sweden is one of the countries where antibiotic prescribing is lowest measured as prescriptions/1000 inhabitants per year [1].

In Sweden the prescribing, especially for respiratory tract infections (RTIs), varies among counties [2], between different primary health care centres (PHCCs) [3], and between individual doctors [4], but there is no medical explanation for the variation [5]. The wide variation in prescribing of antibiotics suggests that guidelines for treatment of common infections are not implemented everywhere.

Strama (the Swedish strategic programme against antibiotic resistance) has worked for a more rational use of antibiotics in Sweden during the last 20 years and through local Strama groups have reduced prescribing of antibiotics both nationally and regionally. Different professions have cooperated in the matter and the prescribing of antibiotics has declined sharply in ambulatory care [2, 6]. Since the year 2000, Strama together with the Medical Product Agency (MPA) have developed guidelines for the diagnosis and treatment of common infections. The guidelines have been disseminated through mailings to the health centres, articles in the Swedish Medical Journal, presentations at national and local meetings and through out-reach visits to the health centres by members of the local Strama groups in each of the 21 counties.

The availability of established guidelines for diagnosis and treatment is seen as a prerequisite for reducing variation in medical practice, especially in modern health care [7]. Modern health care is a complex system, where several factors of importance for the dissemination of knowledge have been identified: the individual professional caregiver, the patient, the professional interaction and organization, including leadership and support systems [7], and it is in the daily conversations that learning and practices develop [8].

In an international perspective, Swedish primary health care has unique features. PHCCs are often organized as group clinics with a limited budget for salaries for all staff, general practitioners (GPs) included, samples and medications. Significant is the close collaboration between nurses and GPs. Telephone counselling is often the first step when the patient needs medical advice or a doctor’s assessment.

Systematic reviews have highlighted factors that may influence the prescribing of antibiotics, for example socio-demographic factors of the doctors, their attitudes, patient characteristics and factors related to health care organization, and they conclude that antibiotic prescribing is a complex process [9, 10]. Near-patient testing has been discussed as a way to reduce uncertainty and improve the physician’s diagnosis in respiratory tract infections in ambulatory care. Near-patient tests are extensively used in Sweden [11] but there are divergent results from studies of their effect on antibiotic prescribing [12–19]. It is unclear how factors interact and how the organization and collaboration within the PHCC affect diagnostic procedures and the prescribing of antibiotics. There are no empirical studies of the interaction between these factors.

The aim of this study was to explore and understand interactions between factors influencing antibiotic prescribing for RTIs in primary care, with special focus on PHCCs with low antibiotic prescribing.

## Methods

During January and February 2014 we studied six PHCCs in three different regions in Sweden. From each region we chose one PHCC with low and one with high prescribing levels compared to the average in the county in the first half of 2013. By studying both types of PHCCs, we expected to gain an understanding of conditions and factors influencing antibiotic prescribing for RTIs, even though the focus of the study was on health centres with low prescribing.

To arrive at an understanding of how these factors interact, we chose a mixed methods design. Our mixed methods design was guided by the Triangulation Design Model according to Creswell both in the design and the analysis of the study [20–22]. The collection of the quantitative and qualitative data was simultaneous and the presentation of the findings from the different sources was integrated in the results and discussion sections. Quantitative data were collected through prescription statistics, questionnaires to patients, and GPs’ audit registrations according to the Audit Project Odense (APO) method [23]. Qualitative data were collected through observations and semi-structured interviews. Observations contribute to visualizing the gap between people’s attitudes and their actions [24, 25]. Observations and interviews were conducted by the same researchers.

**Fig. 1:**
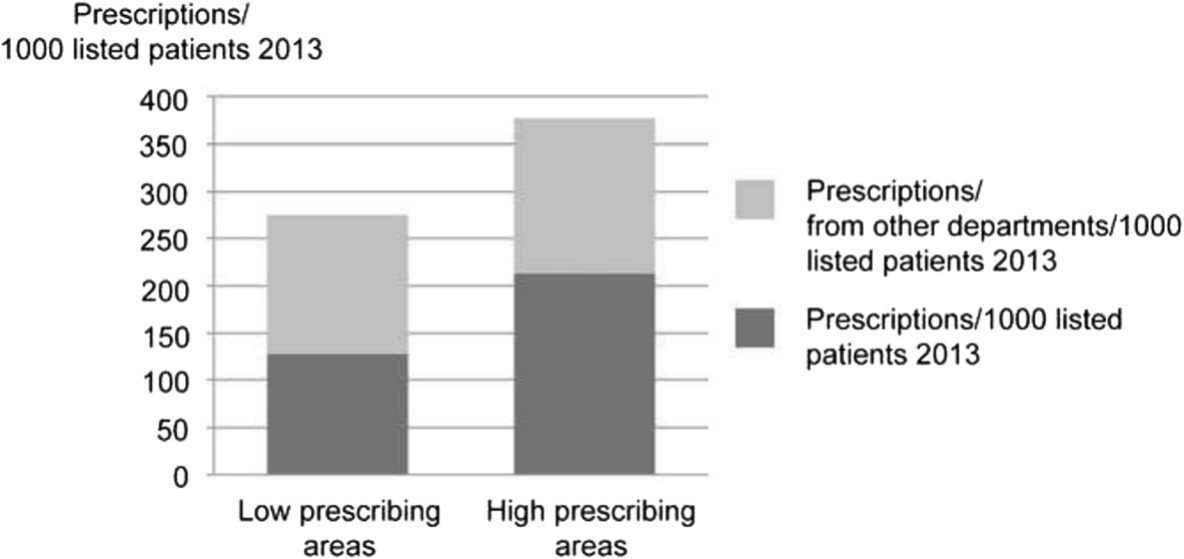
Antibiotic prescriptions. Number of prescriptions/1000 listed patients 2013 in the Primary Health Care Centres

Antibiotic prescription statistics were provided by Apotekens Service AB (Concise) from 2013 and from two weeks before and after the data collection period. The variables studied were antibiotics prescribed by the GPs at the PHCC/1000 listed patients at the PHCC and antibiotics prescribed by physicians from other departments/1000 listed patients at the PHCC (Fig. 1).

A questionnaire survey was performed during the study period. All patients (or legal guardians for children younger than 15 years) with symptoms of RTIs were asked to answer the questionnaire before their consultation with the doctor. Those who had difficulties understanding Swedish were excluded. Questions were asked about age, sex, other diseases, expectations and other questions related to the visit.

Audit registrations were performed by the GPs for each visit for RTIs. Symptoms, sampling, diagnoses and antibiotic treatment as well as the clinical assessment of the severity of the RTI were noted prospectively and consecutively, in accordance with the APO method, which can neither be defined as strictly quantitative nor qualitative. The APO method is a voluntary process with the aim to audit GPs’ own clinical work proceeding from the question, ‘are we doing the right thing in the right way?’.

Observations were performed by six researchers, one GP and one social scientist in each region. During the study period we performed direct observations of the patient’s way through the PHCC. We observed the interaction between patients and employees, the routines of the PHCC concerning RTIs at different work units: telephone counselling, in the reception, the doctor’s surgery, nurse reception, and at different workplace meetings. We also observed the clinical treatment of three different RTI cases: sore throat, pain in the ear and patients with cough where an infection was suspected. All observations were documented as reflective field notes directly after the observation [26].

In the six PHCCs a reasonable proportion of the nurses and GPs and all managers were interviewed. The distribution is shown in Table 1. In all, 53 people were interviewed. Concerning the educational background of the managers, only two were physicians (one operational manager and one senior manager) and the rest were either nurses or had another educational background. Semi-structured interview guides with open-ended questions were used to ensure that all subjects were covered and to stimulate the interviewees’ own narratives. The interviews comprised questions about guidelines, collaboration, education, clinical behaviour including diagnostic procedures, and patient-centred consultation. The predefined categories were derived from the researchers’ preconceptions of the research field. Four researchers were GPs and two were social scientists, five women and one man. The interviews were audio recorded and transcribed verbatim by a professional secretary.

**Table 1:** Interviews - distribution of participants

Profession	Female	Male	n
General Practitioner	11	12	23
Nurse	20	1	21
Operational manager	6	–	6
Senior manager	3	–	3
Total	40	13	53

### Data analysis

The different data were compiled and analysed for each participating PHCC. The quantitative data were analysed by descriptive statistics and the categorical variables were presented as proportions. In comparisons Chi-square test or Fisher’s exact test were used.

For the qualitative data we used qualitative content analysis, an editing analysis style according to Crabtree and Miller [27]. We analysed both the transcribed interviews and the field notes from each observation. The data were organized in categories and themes, predefined as well as newly emerging from the data.

In the next steps, we identified the common traits for the three low-prescribing PHCCs and for the three high-prescribing PHCCs and compared those with each other. This paper presents the characteristics of the low-prescribing PHCCs.

When comparing the PHCCs, the research group used a mixed inductive and deductive approach that built on knowledge gained from the literature on high-performing organizations [28]. The qualitative analysis was performed manually. The results were discussed in the research group until consensus was achieved.

The research team visited each PHCC with feedback on the results of the specific PHCC, which gave all GPs and nurses an opportunity to reflect on the findings, thereby enhancing the validity of our findings.

## Results

The PHCCs selected as low- or high-prescribing PHCCs for the study remained low and high during the course of the study. Figure 1 shows the level of antibiotic prescribing during 2013. Background characteristics of the participating PHCCs are shown in Table 2.

**Table 2:** Description of the Primary Health Care Centres with low and high antibiotic prescribing during 2013

	Low antibiotic prescription PHCC	High antibiotic prescription PHCC
Mean number of GPs (range)	6.4 (4.3–8)	4.4 (2.75–6)
Mean number of nurses (range)	8.6 (5.4–11)	9 (4–16.5)
Mean number of listed patients 31 December 2013	11,400 (7300–14,900)	6900 (3800–8800)
Mean number of telephone instructions/1000 listed patients	3.9	3.1
Mean number of visits/1000 listed patients	1.2	1.5
Mean number of visits/GP	2090	2364
Mean number of listed patients/GP	1765	1568

**Fig. 2:**
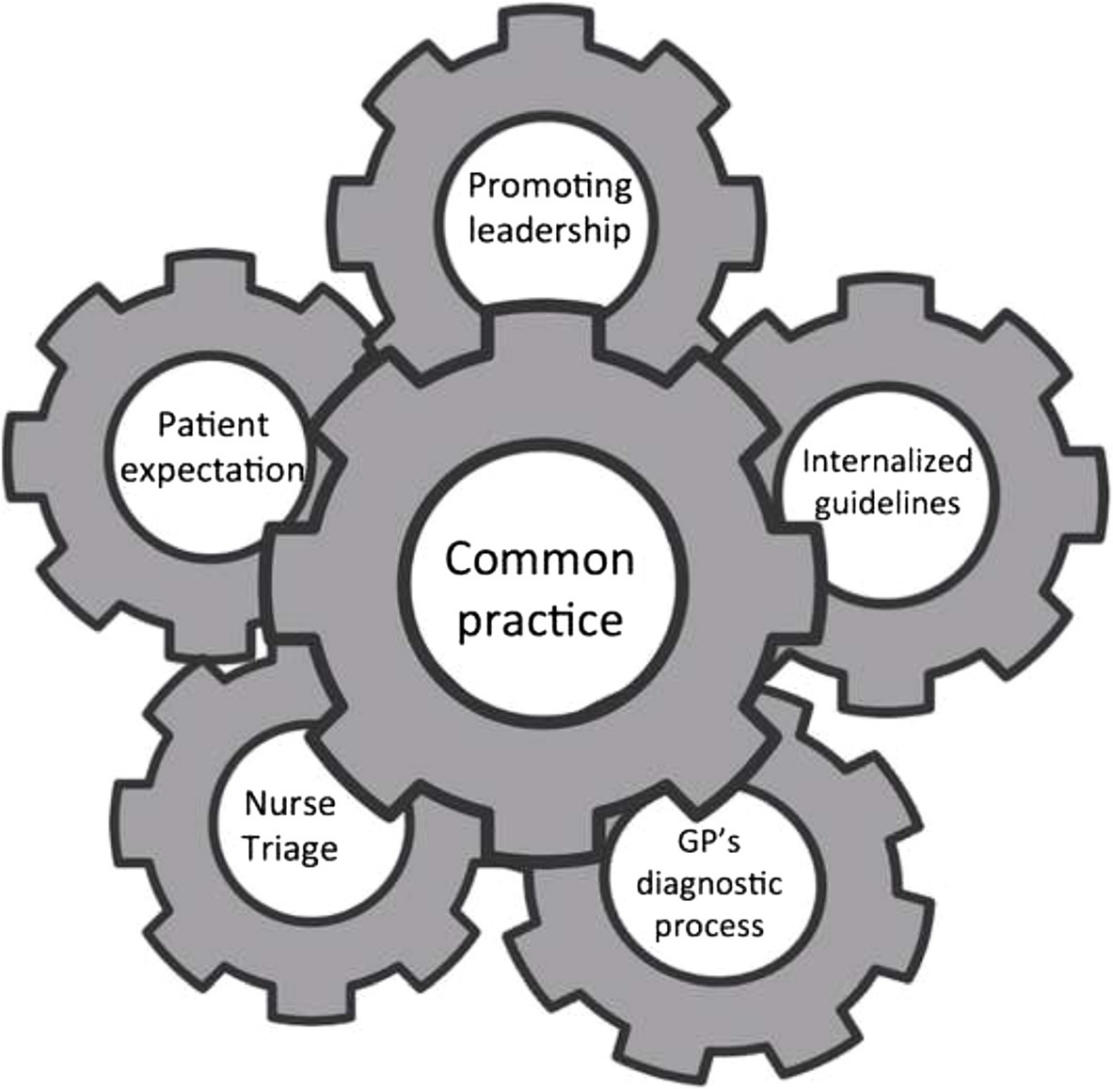
Interacting factors. Important factors and their interactions at Primary Health Care Centres with low antibiotic prescribing for RTIs

The overall theme that characterized PHCCs with low prescribing of antibiotics was *A common practice in line with guidelines* (Fig. 2). We identified five main factors of importance for antibiotic prescribing for RTIs in primary health care: *Promoting management and leadership – facilitating structure*, *Local processing – Internalization of guidelines*, *GP’s diagnostic process*, *Standardized Nurse Triage*, and *Patient expectation* (Table 3).

**Table 3:** Factors influencing antibiotic prescribing – Subcategories, Categories and Theme

Sub categories	Categories	Theme
Dedicated Chief/Medical adviser	Promoting management and leadership – facilitating structure	Common practice in line with guidelines
Promoting evidence-based practice		
The organization’s inner work		
Professional discussions – within and between professions	Local processing – Internalization of guidelines	
Feedback		
Informal opinion moulders		
Testing	GP’s diagnostic process	
Clinical criteria		
Self-care advice	Standardized nurse triage	
Routines and procedures		
Doctor accessibility		
Patient-centred consultation	Patient expectation	
Perceived patient expectation		

### A common practice in line with guidelines

Each main factor was of great importance in the process of achieving a common practice in line with guidelines for RTIs and antibiotic prescribing. The factors could be viewed as cogwheels that lock together in a constant process where internal discussions and continuous professional development activities are 'lubricants' to keep it going. The whole process was supported – and to some extent also controlled – by the manager.

In the three PHCCs with a high prescribing rate and low adherence to guidelines for RTIs, the conditions were reversed. They lacked several of the components demonstrated in Fig. 2, which led to a diverse practice.

#### Promoting management and leadership – facilitating structure

A dedicated leadership played an important role for the PHCC’s ability to achieve and maintain adherence to guidelines for RTIs and antibiotic prescribing. The managers of the low-prescribing PHCCs enabled GPs, nurses and laboratory staff to meet regularly. Professional discussions contributed to integrating new knowledge into practice and also maintaining current knowledge. Both formal and informal leaders took an active part in this process. New colleagues were educated into the culture of the PHCC. Continuous follow-up of prescribing habits was initiated by the operational manager.

“Really important that I am engaged in how we work and that we have good routines and a good structure, so I feel that that is valuable. And I.. what I.. the signals I give to the staff show that I care too about how they prescribe and how they work with infections.” (Manager)

Several GPs expressed a desire to be able to collect data on prescribing themselves. *(Observations, interviews)*

From the observations and interviews we also noted that access to, and encouragement of, regular and planned education was typical for the low-prescribing PHCCs but not for the high-prescribing ones.

#### Local processing – Internalization of guidelines

In PHCCs with low prescribing the guidelines were internalized through an ongoing professional discussion about their content. The process was promoted by formal and informal opinion moulders. As a result all GPs and nurses knew and trusted the guidelines. *(Observations, interviews)*

“But he is after all the one with the knowledge and the authority that he has, and the wisdom too. So it’s like this … everyone listens to what he says, mostly, because he has wise things to say.” (Manager)

Individual feedback on prescribing behaviour, together with professional discussions, was essential both for maintaining and developing a low prescribing of antibiotics for RTIs *(Prescribing statistics, interviews, audit)*. Patients sometimes said one thing to the nurse on the phone and another in the personal meeting with the GP. Feedback between GPs and nurses on the severity of the infection seemed more frequent in the low-prescribing PHCCs *(Observations, interviews)*.

#### GP’s diagnostic process

In low-prescribing PHCCs the GP decided whether tests should be taken or not. When the GP considers antibiotic treatment for e.g. sore throat the guidelines stipulate a confirmation by taking near-patient rapid antigen detection test (RADT). The GPs said that they were aware of that particular guideline.

“Well, it has been said … if we are to consider treatment, you must have a positive RADT too.” (Doctor)

Near-patient tests, in particular C-reactive protein (CRP), were used less frequently by the GPs at the low-prescribing PHCCs. GPs at low-prescribing PHCCs clearly expressed that clinical criteria need to be present e.g. to take RADT. *(Observations, interviews, audit)*

“I never take a RADT without having set up these criteria first, you know, because taking a RADT with no grounds, that’s totally crazy.” (Doctor)

According to the audit registration, GPs at low-prescribing PHCCs prescribed fewer antibiotics also during the study period and GPs in high-prescribing PHCCs used near-patient tests more frequently. The latter also reported more frequent use of clinical assessment alone, without acknowledged diagnostic criteria. Almost every infection was classified as mild or moderate by the GP, but more were classified as mild among the low-prescribing and more were classified as moderate among the high-prescribing PHCCs. A larger proportion of the infections were diagnosed as upper respiratory tract infection at the low-prescribing and as pneumonia at the high-prescribing PHCCs (Table 4) *(Audit)*.

**Table 4:** The GP’s assessment of the severity of the infection, near-patient testing, antibiotic prescriptions and diagnoses

	Number (percentage)		
	Low antibiotic prescription PHCC	High antibiotic prescription PHCC	
	*n* = 128	*n* = 126	p*
Women	77 (60.6)	60 (47.6)	*0.04*
*Assessment of the severity of the infection*			
Mild infection	82 (72.6)	63 (54.3)	*0.14*
Moderate infection	29 (25.7)	51 (44.0)	
Severe infection	2 (1.8)	2 (1.7)	
*Performed testing*			
CRP	35 (28.9)	67 (53.2)	*<0.001*
RADT – Strep A	18 (15.4)	31 (27.9)	*0.021*
Other testing	8 (10.0)	13 (33.3)	*0.002*
Any testing	56 (43.8)	100 (79.4)	*<0.001*
*Diagnoses*			
Common cold	68 (53.1)	47 (37.3)	*0.011*
Acute media otitis	23 (18.0)	15 (11.9)	0.18
Sinusitis	2 (1.6)	6 (4.8)	0.14
Tonsillitis	11 (8.6)	20 (15.7)	0.08
Acute bronchitis	11 (8.6)	10 (7.9)	0.85
Pneumonia	2 (1.6)	15 (11.9)	*0.001*
Other infection	14 (10.9)	14 (11.1)	0.97
Antibiotic prescription	31 (24.6)	46 (36.8)	*0.04*

*Comparisons are made by Chi-squared test or Fisher’s exact test (F)

#### Standardized nurse triage

Telephone triage by a nurse or sometimes also by a doctor was a prioritized task at PHCCs where prescribing of antibiotics was low. Characteristic for such PHCCs was that there were special routines for nurse triage. Patients were passed through after a first contact with a nurse, often by telephone. When a patient or a parent called the PHCC for an RTI, the nurse had an important role assessing whether the patient could manage with self-care advice or if a GP consultation was necessary. If the nurse believed that self-care advice was not enough, the nurse gave the patient a consultation with one of the permanent doctors and not the locums. *(Observations, interviews)*

Where the guidelines were clear and implemented and internalized, the nurse had a relatively easy task to decide whether a GP consultation was necessary, even if there always was a certain uncertainty and sometimes even fear of making mistakes:

“our role is rather important, precisely to inform about self-care in situations where self-care is the thing to do and there both the triage handbook and the Strama guidelines are fairly clear and easy to follow, but of course there are exceptions where you … and then I think that children are the most difficult and elderly people with multiple diseases of course too.” (Nurse)

Routines and procedures must be clear enough for the nurse or the GP to make the right decisions about which patient needs an appointment:

“But there the nurse has an important role in being able, well, at least making sure that only those come … that it’s the right patients who come, that those who don’t need to come don’t come.” (Manager)

Patients who were examined at the high-prescribing PHCCs reported to a greater extent that it was easy to get an appointment with the GP (Table 5).

**Table 5:** Patients’ reports of knowledge, duration of symptoms and expectations

	Low antibiotic prescribing PHCC	High antibiotic prescribing PHCC	
	*n* = 71 (%)	*n* = 120 (%)	p*
Children	19 (30.2)	39 (35.5)	0.48
Women	50 (74.6)	75 (64.1)	0.14
It was easy to get an appointment with the GP?	60 (85.7)	112 (96.6)	*0.007*
I know if I need an antibiotic prescription	23 (33.8)	28 (24.1)	0.16
Have confidence in your doctor’s decision not to prescribe antibiotics	42 (62.7)	79 (68.7)	0.41
Antibiotics help against bacteria	50 (73.5)	78 (67.8)	0.42
Antibiotics help against virus	30 (44.1)	47 (40.9)	0.67
Antibiotics speed the healing of cold	33 (49.2)	60 (53.6)	0.58
Coloured mucus needs antibiotics	42 (61.8)	66 (57.9)	0.61
Cough that has lasted long needs antibiotics	43 (63.2)	68 (59.6)	0.63
*How long have you had trouble?*			
<3 days	19 (27.5)	29 (25.0)	0.75
4–7 days	16 (23.2)	35 (30.2)	
>1 week < 3	13 (18.8)	24 (20.7)	
3 weeks or more	21 (30.4)	28 (24.1)	
*Expectation of today’s visit*			
get an evaluation	53 (74.6)	93 (77.5)	0.65
get advice	29 (40.8)	45 (37.5)	0.65
that tests are taken	26 (36.6)	40 (33.3)	0.64
get something soothing	32 (45.1)	46 (38.3)	0.36
get antibiotics	22 (31.0)	21 (17.5)	*0.03*
get sick leave	4 (5.6)	10 (8.3)	0.49

*Comparisons are made with Chi-squared test

#### Patient expectation

In PHCCs where prescribing of antibiotics was low, nurses and GPs expressed that patients do not want unnecessary antibiotic treatments but rather good advice about how to treat the infection. Of all patients, 24% were expecting to receive an antibiotic prescription. Significantly more of these were visiting the low-prescribing PHCCs. Most of the patients answering the questionnaire knew that antibiotics were effective against bacteria and almost half of them thought they were effective against viruses (Table 5). *(Prescribing statistics, interviews, audit, patient survey)*

The GPs at the low-prescribing PHCCs started the consultations in a more patient-centred way with open-ended questions in order to get a picture of the patients’ expectations. They only introduced antibiotics when they had clinically assessed all circumstances and had come to the conclusion that an antibiotic treatment was needed according to the guidelines. Nurses also said that they had observed a change in patients’ expectations in this direction over the past five years or so. *(Observations, interviews)*

“people are so aware today that you shouldn’t take it [antibiotics] unnecessarily and the development of resistance and so on, so I don’t think so. We don’t have that type of patients here who demand antibiotics, I don’t think so.” (Nurse)

## Discussion

From the analysis of the data from the different sources an overall theme emerged: A common practice in the PHCC is crucial for low antibiotic prescribing in line with guidelines. Several factors contribute such as promoting management and leadership, internalized guidelines, the GP’s diagnostic process, nurse triage, and patient expectation. These factors were closely related and influenced each other.

The results also showed that new knowledge was internalized both in the PHCC as a whole and in all members of the staff. The guidelines were normative for the group as well as for every individual in the group.

### Methodological considerations

The mixed methods design enabled us to get as complete a picture as possible of the studied PHCCs. Six PHCCs might seem too few, but with the mixed methods design we have obtained abundant data. One difficulty was to get an overview of all research data. A remedy was separate steps of the analysis and the fact that the researchers had different research experience. The analysis with both inductive and deductive elements as well as the descriptive statistics from the patient survey and the audit increased the validity of the results.

The observations gave an idea of the local culture at the PHCC as well as relations and interaction between the employees. Through the observations we reached an understanding of what actually happened between the patients and the staff on their way through the PHCC, although compared to ethnographic research the study period was relatively short. We believe this was outweighed by the presence of more than one observer.

The study period would normally have been a high season for respiratory tract infections, but during 2014 the infection season appeared later than usual and therefore there were fewer patients with RTIs than expected. Additionally, there were sometimes difficulties convincing the patients about the importance of answering the questionnaire and sometimes the receptionists forgot or did not have the time to hand them out. Due to the low numbers in both the patient survey and the audit, the data must be interpreted with caution when standing alone, but when aggregated with data from the interviews and observations the findings were strengthened.

We focused specifically on what happened at the low-prescribing PHCCs, even though we also collected data from high-prescribing PHCCs. Those were collected in order to mirror the low-prescribing PHCCs and to achieve a better understanding of factors influencing antibiotic prescribing for RTIs in primary health care.

The organization of Swedish PHCCs as group clinics implies that the findings of this study must be interpreted with reservations in an international perspective where group clinics are less common.

### Comparison with existing literature

#### A common practice

Previous studies have shown that new knowledge will be used in clinical practice, when transformed through continuous discussions among staff. New knowledge must be applicable and meaningful in the local context and its credibility is tested in a learning process [8, 29]. Such a process requires forums and opportunities for discussions. Low-prescribing PHCCs had set aside time for regular meetings for medical issues and prioritized training, within and between professions, which enabled this process.

This study identified the leadership as a crucial factor for low antibiotic prescribing. The leadership must provide structural conditions and encouragement for inter-professional collaboration in order to achieve a common practice in keeping with guidelines. This is in line with the conclusions of two recent studies [28, 30]. In the present study the management strove for a common goal using feedback and active discussion regarding antibiotic prescribing. At low-prescribing PHCCs, GPs were also acting as explicit local opinion moulders, an important function for knowledge translation [31]. Organizational readiness for change is also of importance. Unfortunately according to a systematic review from 2014 [32], practically no instruments have been developed for assessing organizational readiness for knowledge translation in health care. Developing such instruments might be a subject for future research. Our findings strengthen that the Strama model is a successful way of working. Strama contributes to enhanced discussions among different professions about antibiotic use for RTIs and supports the importance of feedback. Feedback helps to show that you do not always do what you think and therefore can stimulate behaviour change [31].

In our study, all PHCCs were given feedback on the health centre’s prescribed antibiotics every three months. At the low-prescribing PHCCs these data were discussed at staff meetings. According to the interviews, GPs would need easier access to a relevant presentation of their own prescribing data.

Several reviews have stressed well-functioning inter-professional collaboration as an important condition for knowledge dissemination and evidence-based practice [7, 33]. In this study the collaboration between nurses and GPs appeared particularly important. The nurses’ triage to either self-care or appointment to the GP was decisive for which patients were offered an appointment. Some studies have shown that fruitful collaboration between GPs and nurses is based on professional respect and confidence in each other’s professional competence [34, 35]. These conditions were present in the low-prescribing PHCCs. There was also a consensus that most infections are self-limiting and for those patients self-care was the first choice. Consequently these patients, not in need of an appointment, escape unnecessary testing and antibiotic treatment. Thus, correct triage is an important factor behind low antibiotic prescribing.

The patients had generally good knowledge of antibiotics. Most of the patients answering the questionnaire knew that antibiotics were effective against bacteria and less than half of them thought they were effective against viruses. This proportion is in line with an earlier Swedish study and better than in a Spanish study [36, 37].

More patients in the low-prescribing PHCCs expected antibiotics compared to patients in the high-prescribing PHCCs. A probable explanation might be that the nurses at the low-prescribing PHCCs had selected patients with respiratory infections who might benefit from antibiotic treatment according to guidelines. However, the GPs at low-prescribing PHCCs rated most infections as mild while GPs at high-prescribing PHCCs more often classified the respiratory tract infection as moderate. The more common use of bacterial diagnoses among the high prescribers compared to low prescribers has been shown earlier [38, 39]. The different assessment of the severity of the infection in our study, together with the different proportions of diagnoses requiring antibiotic treatment, may thus be seen as a way to retrospectively legitimize prescribing of antibiotics. The individual prescribing patterns may have greater significance than the clinical picture for treatment decisions, consistent with previous studies [40]. In our study, one third of the patients were treated with antibiotics, which is lower than in most international studies [38, 41].

There has been a paradigm shift regarding the management of infections over the past 20 years, from an earlier approach according to which all infections of probable bacterial aetiology had to be treated with antibiotics, to a more modern policy of treating with antibiotics only if there is evidence for benefit [42, 43]. The earlier approach reappeared in several interviews, especially in the high-prescribing PHCCs. Moreover, our study showed that high-prescribing PHCCs also used near-patient tests (both RADT and CRP) to a higher degree than low-prescribing PHCCs. Thus, high use of rapid near-patient tests may be associated with high prescribing of antibiotics. This result differs from previous studies where the use of antibiotics decreased after these tests were introduced [16]. The differences may be due to a different prescribing pattern than in Sweden, or that the tests were introduced with clear and specific guidelines for their use. The result in our study is in line with others indicating that the use of RADT and CRP not according to guidelines increases the risk of overtreatment with antibiotics [11–15].

## Conclusions

In PHCC with low antibiotic prescribing some important factors were observed. Guidelines were adapted and transformed into a common practice within the staff at the PHCC, not only the GPs. Nurses’ triage in the telephone played an important role in daily practice. Encouragement from the manager stimulated inter-professional discussions concerning antibiotic prescribing and informal opinion moulders talking about antibiotic prescribing were supported by the managers. Near-patient tests were used in accordance with the guidelines. Moreover, routines and IT systems were adapted in a way that enabled GPs themselves to access prescribing data easily from registers as a basis for professional discussions. Finally, continuous professional development activities were encouraged for everyone to be able to have up-to-date knowledge. All these factors were interacting in the PHCC with low antibiotic prescribing and the managers need to facilitate them. The factors also have to be taken into account when making interventions to rational antibiotic use.

## Abbreviations

APO, Audit Project Odense; CRP, C-Reactive Protein; GP, General Practitioner; MPA, Medical Product Agency; PHCC, Primary Health Care Centre; RADT, Rapid Antigen Detection Test; RTI, Respiratory Tract Infection; Strama, Swedish strategic programme against antibiotic resistance
